# Supplementary material for: Resolving the full spectrum of human genome variation using Linked-Reads
Source: Genome Res. 2019 Apr;29(4):635–45. doi: 10.1101/gr.234443.118 (PMC6442396; doi:10.1101/gr.234443.118)
Supplement: Supplemental Material [file supp_gr.234443.118_Supplemental_longranger-2.2.zip › longranger-2.2.tar.bz2!/lr2.2-public/lib/stan/cmdstan/stan/doc/api/html/math_2error__handling_8hpp.html]

Stan: src/stan/math/error\_handling.hpp File Reference


|  |  |
| --- | --- |
|  | Stan  2.5.0  probability, sampling & optimization |


- Main Page
- Related Pages
- Namespaces
- Classes
- Files

- File List
- File Members

All Classes Namespaces Files Functions Variables Typedefs Enumerations Enumerator Friends Macros Pages

- src
- stan
- math

error\_handling.hpp File Reference

`#include <algorithm>`  
`#include <cstddef>`  
`#include <limits>`  
`#include <utility>`  
`#include <iostream>`  
`#include <boost/type_traits/is_unsigned.hpp>`  
`#include <stan/meta/traits.hpp>`  
`#include <stan/math/error_handling/dom_err.hpp>`  
`#include <stan/math/error_handling/dom_err_vec.hpp>`  
`#include <stan/math/error_handling/check_not_nan.hpp>`  
`#include <stan/math/error_handling/check_equal.hpp>`  
`#include <stan/math/error_handling/check_finite.hpp>`  
`#include <stan/math/error_handling/check_greater.hpp>`  
`#include <stan/math/error_handling/check_greater_or_equal.hpp>`  
`#include <stan/math/error_handling/check_less.hpp>`  
`#include <stan/math/error_handling/check_less_or_equal.hpp>`  
`#include <stan/math/error_handling/check_bounded.hpp>`  
`#include <stan/math/error_handling/check_nonnegative.hpp>`  
`#include <stan/math/error_handling/check_positive.hpp>`  
`#include <stan/math/error_handling/check_positive_finite.hpp>`  
`#include <stan/math/error_handling/check_consistent_size.hpp>`  
`#include <stan/math/error_handling/check_consistent_sizes.hpp>`

Go to the source code of this file.


---

[ Stan Home Page ]

*© 2011–2014,
Stan Development Team.*
